# Lipid droplet associated protein HILPDA promotes hypoxia-induced ferroptosis by driving LPCAT3-mediated polyunsaturated phospholipids enrichment

**DOI:** 10.1371/journal.pone.0350129

**Published:** 2026-06-08

**Authors:** Zhenmei Song, Wenli Li, Jie Zeng, Xuexin Wang, Dezhi Wang, Xingchen Liao

**Affiliations:** 1 Department of Gastroenterology, China-Japan Friendship Hospital, Beijing, China; 2 Department of Rheumatology, China-Japan Friendship Hospital, Beijing, China; 3 Department of Urology, The Second Affiliated Hospital, School of Medicine, South China University of Technology, Guangzhou, China; 4 Medical School of Chinese PLA, Chinese PLA General Hospital, Beijing, China; 5 Department of Gastroenterology, The Seventh Medical Center of Chinese PLA General Hospital, Beijing, China; Sun Yat-Sen University, CHINA

## Abstract

Each year, tens of millions of individuals sojourn to high-altitude environments (>2500 m), where they face the significant physiological challenge of hypoxia, a condition that often induces a range of gastrointestinal disorders. While our prior studies linked this damage to ferroptosis, the role of hypoxia-inducible lipid droplet-associated protein (HILPDA), a hypoxia inducible factor-1α/2α (HIF-1α/2α) downstream regulator, in this process remains unclear. This study aimed to explore the role and mechanism of HILPDA in regulating ferroptosis of normal human gastric and small intestinal epithelial cells (NGEC and HIEC) under hypoxic conditions. Our results found that overexpression of HIF-1α, HIF-2α, and HILPDA exacerbated hypoxia-induced cell death, which was reversed by the ferroptosis inhibitor ferrostatin-1. Knockdown of HIF-1α/2α inhibited HILPDA expression. Furthermore, knockdown of HILPDA led to a reduction of the hypoxia-induced lipid peroxidation, and the ferroptotic characteristics of cellular mitochondria observed under transmission electron microscopy. Conversely, HILPDA overexpression reversed the protective effects of HIF-1α/2α knockdown. Lipidomic analysis further revealed that HILPDA knockdown significantly decreased the levels of polyunsaturated fatty acid-phosphatidylcholines (PUFA-PCs) and phosphatidylethanolamines (PEs) under hypoxia. Compared to HIF-1α knockdown, HILPDA knockdown led to a slight difference in PUFA-PEs without significant difference in PCs and phosphatidylinositols (PIs) under hypoxia. Compared to HIF-2α knockdown, HILPDA knockdown resulted in negligible differences in PEs, PCs, and PIs. In addition, HILPDA knockdown downregulated Lysophosphatidylcholine Acyltransferase 3 (LPCAT3), and overexpression of LPCAT3 significantly attenuated the inhibitory effect of HILPDA knockdown on hypoxia-induced ferroptosis. In conclusion, HILPDA enhanced susceptibility to hypoxia-induced ferroptosis in NGEC and HIEC by enriching PUFA-containing phospholipids through LPCAT3. These findings identified the HIF-1α/2α-HILPDA-LPCAT3 axis as a pivotal pathway driving hypoxia-induced ferroptosis, therefore providing potential therapeutic targets for gastric and small intestinal mucosal injury associated with hypoxia.

## Introduction

Tens of millions of people travel to high-altitude regions (>2500 m) annually for tourism, pilgrimage, or work, where they face the significant physiological challenge of hypoxia. This condition frequently leads to gastrointestinal disorders such as anorexia, nausea, diarrhea, vomiting, and bleeding, which predominantly affect the upper gastrointestinal tract [1–4]. Cellular adaptation to hypoxia is a fundamental biological process governed by hypoxia-inducible factor (HIF), mainly HIF-1α and HIF-2α, which orchestrate transcriptional programs encompassing angiogenesis, glycolysis, and erythropoiesis, enabling tissues to survive under low oxygen conditions [5]. However, prolonged or severe hypoxia can shift balance toward regulated cell death pathways [6].

Ferroptosis—an iron-dependent form of cell death driven by lipid peroxidation—occurs in hypoxic cells [7]. The lack of oxygen triggers a state of oxidative stress, compelling cells to generate excessive reactive oxygen species, which in turn initiates ferroptosis [8]. It has been established that HIF-1α/2α plays a role in modulating this process. For example, suppressing HIF-1α has been shown to mitigate ferroptosis in knee osteoarthritis [9]. Similarly, inhibition of HIF-1α/2α alleviates hypoxia-induced ferroptosis in gastrointestinal mucosal [10]. Central to ferroptosis is the peroxidation of polyunsaturated fatty acid-phospholipids (PUFA-PLs), which compromises the integrity of cellular membranes, rendering them vulnerable to oxidative damage [11,12]. Recent studies have highlighted the role of lipid metabolic enzymes, such as acyl-CoA synthetase long-chain family member 4 (ACSL4), in determining a cell’s susceptibility to ferroptosis by modulating the incorporation of PUFAs into membranes [13]. ACSL4 catalyzes the activation of free polyunsaturated fatty acids (PUFAs) to their corresponding acyl-CoA esters (PUFA-CoAs) [14]. Beyond ACSL4, the remodeling of membrane phospholipids is also critically dependent on enzymes like lysophosphatidylcholine acyltransferase 3 (LPCAT3). LPCAT3 preferentially incorporates PUFAs into the sn-2 position of lysophospholipids, particularly phosphatidylcholines (PCs) and phosphatidylethanolamines (PEs) [15], thereby enriching the cellular membrane with substrates prone to peroxidation [16]. Consequently, LPCAT3 has been identified as a key determinant of ferroptosis sensitivity [17]. In contrast, monounsaturated fatty acids (MUFAs) can physically displace more oxidizable PUFAs from PLs, thus creating a membrane environment less prone to lipid peroxidation [18].

Emerging evidence suggests that hypoxia and HIF-1α could regulate ferroptosis through the modulation of LPCAT3. For instance, in prostate cancer, hypoxia induces lipid droplet accumulation and LPCAT3 expression, reduces PEs levels, thereby promoting resistance to ferroptosis [19]. However, in acute pancreatitis, HIF-1α upregulates LPCAT3 expression to promote ferroptosis [20]. Similarly, hypoxic exposure directly induces LPCAT3 expression and subsequent ferroptosis in small glial cells [21].

It has been well documented that hypoxia alters lipid metabolism, stimulating the synthesis of PUFAs and the formation of lipid droplets [22], partly by upregulating hypoxia-inducible lipid droplet-associated protein (HILPDA), a HIF-1α/2α target gene involved in lipid storage and lipolysis [23]. Specifically, HIF-1α transcriptionally activates HILPDA by binding to hypoxia-responsive elements in its promoter region [24]. Meanwhile, HIF-2α increases the expression of perilipin 2 and HILPDA, leading to selective accumulation of PUFAs, heightened oxidative stress, and subsequent ferroptosis [25,26]. Notably, HILPDA enriches the levels of PUFA-phosphatidylethanolamines (PEs) and PUFA- triacylglycerols, thereby increasing the sensitivity of renal clear cell carcinoma to ferroptosis [25]. The activation of the HIF-2α/HILPDA pathway promotes the accumulation of PUFA-enriched triglycerides and cholesteryl esters within lipid droplets, which enhances membrane PLs with PUFAs through lipolysis and lipophagy, exacerbating lipid peroxidation and sensitizing cells to ferroptosis [27].

However, the role of HILPDA in lipid metabolism and ferroptosis is not uniformly consistent. For instance, studies in colon cancer cells have demonstrated that HILPDA knockdown significantly increases the abundance of phosphatidylcholines (PCs), PEs, and phosphatidylinositols (PIs) under hypoxic conditions [28]. Furthermore, HILPDA deficiency has been observed to disrupt lipid homeostasis and increases PUFA enrichment in membrane PLs in hepatocellular carcinoma cells [29].

Our previous study revealed that hypoxia led to gastric and small intestinal mucosal damage, specifically through HIF-1α/2α-mediated ferroptosis, rather than through apoptosis, necrosis, or pyroptosis [10,30]. Furthermore, it was determined that ferroptosis was achieved by promoting HIF-1α/2α-mediated ALOX5, NADPH Oxidase 4 (NOX4), and PUFA-PLs [10]. Specifically, ALOX5 catalyzes the peroxidation of PUFA-PLs, while NOX4 exacerbates oxidative stress, collectively creating a pro-ferroptotic microenvironment. These findings aligned with evidence that HIF-2α/HILPDA signaling promoted PUFA-enriched PLs, thereby exacerbating lipid peroxidation.

Despite these insights, the precise role of HILPDA in hypoxic gastrointestinal epithelial cells, particularly its influence on lipid remodeling and ferroptotic susceptibility, remained poorly defined. Therefore, this study aimed to investigate the effects and underlying mechanisms of HILPDA in ferroptosis within hypoxic gastrointestinal mucosal epithelia.

## Materials and methods

### Cell culture and transfection

Normal human gastric epithelial cell (NGEC) and Normal human small intestinal epithelial cell (HIEC) were obtained as described previously [10]. The cells were cultured in Dulbecco Modified Eagle medium containing 10% (v/v) fetal bovine serum and 1% (v/v) penicillin/streptomycin, within a standard cell culture incubator at 37°C under normoxic conditions (21% O_2_ and 5% CO_2_). To perform hypoxic experiments, the cells were incubated in a hypoxic chamber, maintaining an atmosphere of 1% O_2_ and 5% CO_2_. For overexpression transfection, vector plasmid, HIF-1α plasmid, HIF-2α plasmid, or HILPDA plasmid were transfected into the cells under normoxia for 24 h. The cells were then placed in hypoxia for 24 h and treated with dimethyl sulfoxide or 5μM ferrostatin-1 (Fer-1) for another 24 h. Subsequently, the cells were collected for crystal violet staining, trypan blue staining and cell counting kit-8 (CCK-8) assay. For knockdown experiments, siRNAs targeting negative control (NC), HIF-1α, HIF-2α, or HILPDA were transfected into the cells under normoxic conditions for 24 h, after which the cells were exposed to hypoxia for 48 h. The cells were then harvested for western blot, lipid peroxidation detection, transmission electron microscopy (TEM), and lipidomic analysis. For co-transfection experiments, HIEC was transfected with siRNAs targeting HIF-1α or HIF-2α (or a non-targeting control, siNC) together with either a HILPDA overexpression plasmid or an empty vector. After 24 h under normoxia, cells were subjected to 48 h of hypoxia and subsequently harvested for analysis of cell death (trypan blue staining) and lipid peroxidation (4-Hydroxynonenal (4-HNE) and malondialdehyde (MDA)). Separately, HIEC was co-transfected with siRNAs targeting HILPDA or a non-targeting control along with either a LPCAT3 overexpression plasmid or an empty vector for 24 h under normoxia, after which the cells were exposed to hypoxia for 48 h and subsequently harvested for trypan blue staining and MDA assay.

### Crystal violet staining

The medium was carefully aspirated from the 12-well plates, and the cells were washed three times with phosphate-buffered saline (PBS) and fixed with 4% paraformaldehyde for 40 min. Following fixation, the paraformaldehyde was thoroughly removed, and the cells were stained with a crystal violet solution (C0121, Beyotime Biotechnology, Shanghai, China) for 30 min, and then the crystal violet solution was removed and the wells were washed with PBS.

### CCK-8 assay

Cells in a 96-well plate were added with 10uL of CCK-8 reagent (CK04, Dojindo Laboratories, Kumamoto, Japan) per well. Absorbance at 450nm was measured after one hour.

### Trypan blue staining

An aliquot (10 μL) of the cell suspension was combined with an equal volume of trypan blue staining solution (C1313S; Beyotime Biotechnology) in a centrifuge tube and mixed thoroughly. The mixture was loaded into a hemocytometer and analyzed using an automated cell counter (Countstar Mira BF; Ruiyu Biotech, Shanghai, China). The cell death rate is determined by the percentage of trypan blue staining-positive cells relative to the total number of cells.

### Western blot

Total protein was extracted using RIPA buffer and quantified using the BCA assay. Equal amounts of protein were separated by SDS-PAGE on 12% gels and transferred onto a nitrocellulose membrane. The membrane was incubated with primary antibodies targeting HIF-1α (1:1000; ab179483, Abcam, Waltham, MA, USA), HIF-2α (1:1000; PA1–16510, Invitrogen, Carlsbad, CA, USA), HILPDA (1:1000; GTX16280, GeneTex, San Antonio, TX, USA), LPCAT3 (1:1000, HA723171, HUABIO, Hangzhou, Zhejiang, China), and GAPDH (1:10000; ab8245, Abcam) followed by horseradish peroxidase (HRP)-conjugated anti-mouse or anti-rabbit secondary antibodies (1:10000; ZB-2301 and ZB-2305, ZSGB-Bio, Beijing, China). Protein bands were detected using a gel imaging system from SinSage Technology Co., Ltd. (Beijing, China).

### Detection of 4-HNE

The level of 4-HNE was measured using a competitive enzyme-linked immunosorbent assay (E-EL-0128, Elabscience, Wuhan, China). The detailed procedure was as follows: cellular samples were washed with PBS, subjected to repeated freeze-thaw cycles for lysis, and centrifuged to obtain the supernatant. In the assay, standards and samples were added to the pre-coated microplate wells, followed by the addition of biotinylated detection antibody working solution, and incubated at 37°C for 45 min. After washing, HRP conjugate working solution was added and incubated at 37°C for 30 min, followed by another washing step. Then, 3,3,’5,5’-Tetramethylbenzidine substrate was added and incubated in the dark for 15 min at 37°C. Finally, the reaction was stopped, and the absorbance was measured at 450 nm.

### Detection of MDA

The concentration of MDA was determined using a commercial assay kit (E-BC-K028-M, Elabscience). Briefly, after cell collection and removal of the supernatant, 0.5 mL of extraction solution was added to the cell pellet, followed by ultrasonication to obtain a homogeneous suspension. For the assay, blank tubes containing 0.1 mL of absolute ethanol, standard tubes with 0.1 mL of 10 nmol/mL standard, and sample tubes with 0.1 mL of cell suspension were prepared. Each tube was mixed with 1 mL of working solution, securely sealed, and incubated in a 100°C water bath for 40 min. After cooling under running water and centrifugation, 0.25 mL of the supernatant was carefully transferred to a microplate, and absorbance was measured at 532 nm.

### TEM

Samples were fixed in 2.5% glutaraldehyde at 4°C, post-fixed in 1% osmium tetroxide, dehydrated in a graded series of ethanol, permeabilized in acetone: epoxy mixtures (2:1, then 1:1), and embedded in epoxy. Thin sections (80–100 nm) were prepared using an ultramicrotome and then stained with 2% uranyl acetate and lead citrate before imaging by a transmission electron microscope (TECNAI G 20 TWIN, FEI Company, Hillsboro, State of Oregon, USA). To provide a quantitative assessment of mitochondrial exhibiting features of ferroptosis, a systematic analysis based on TEM images was performed. For each experimental group, three randomly selected TEM images were analyzed. In each image, the total number of mitochondria was counted, and mitochondria displaying characteristic ferroptotic features (including shrinkage, increased membrane density, and reduced or absent cristae) were identified. The percentage of mitochondria exhibiting ferroptosis characteristics was calculated per image, and the mean percentage ± SEM was determined for each group.

### Lipidomic analysis

The lipidomic analysis was performed as described in our previous study [10].

### Statistics analysis

Data were assessed using GraphPad Prism 9.0. Comparisons between two groups were performed using Student’s *t*-test and comparisons among more than two groups were performed using one-way analysis of variance. A significance level of *p* < 0.05 was considered statistically significant. Results are presented as mean ± standard error of the mean.

## Results

### Overexpression of HIF-1α/2α and HILPDA exacerbated cellular ferroptosis induced by hypoxia

**Fig 1:**
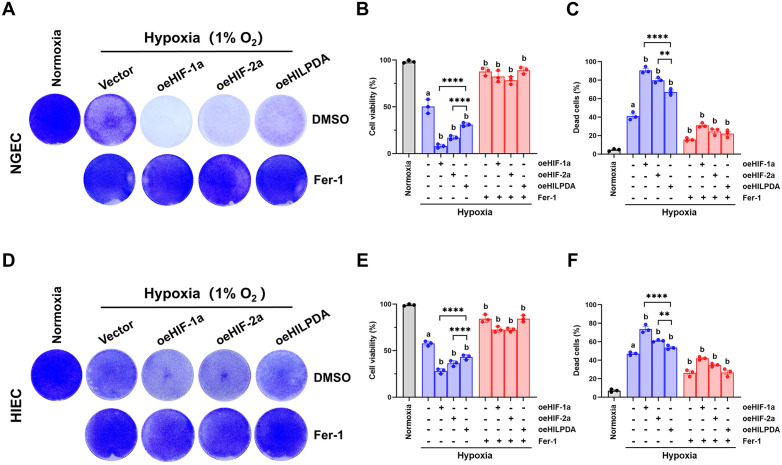
Overexpression of HIF-1α/2α and HILPDA exacerbated cellular ferroptosis induced by hypoxia. Empty vector plasmid, HIF-1α plasmid, HIF-2α plasmid, or HILPDA plasmid was transfected into NGEC and HIEC under normoxia for 24 h. The cells were then placed in hypoxia (1% O_2_) for 24 h and treated with dimethyl sulfoxide (DMSO) or 5μM ferrostatin-1 (Fer-1) for another 24 h. Cell viability by using crystal violet staining (A, D), CCK-8 assay (B, E), and trypan blue staining (C, F). Hypoxia + vector group vs. Normoxia control, ^a^ *p*<0.0001 in B, C, E, and F. Hypoxia + HIF-1α, hypoxia + HIF-2α, hypoxia + HILPDA, Hypoxia + HIF-1α + Fer-1, hypoxia + HIF-2α + Fer-1, or hypoxia +HILPDA + Fer-1 group vs. hypoxia + vector group, ^b^ *p*<0.05 at least in B, C, E, and F. The data are presented as the mean ± standard errors of the mean. A representative of at least three independent experiments is shown. **p<0.01 and ****p<0.0001.

Based on our previous studies demonstrating that hypoxia-induced cell death in gastric and intestinal mucosal cells was predominantly ferroptosis and was mediated by HIF-1α/2α [10,30], the present study aimed to investigate whether HILPDA—which is also upregulated under hypoxic conditions—played a functional role in this HIF-1α/2α-driven ferroptotic pathway or merely represented a bystander phenomenon. To address this question, we initially overexpressed HIF-1α, HIF-2α, or HILPDA to determine the contribution of HILPDA and to compare its effect with that of HIF-1α and HIF-2α on ferroptosis susceptibility. The effects of overexpression of HIF-1α, HIF-2α, and HILPDA on cell survival under hypoxic conditions were detected by crystal violet staining, CCK-8 assay, and trypan blue staining. The results showed that overexpression of HIF-1α and HIF-2α significantly exacerbated hypoxia-induced cell death compared to the control group (Fig 1). Notably, while overexpression of HILPDA also exacerbated cell death, its effect was weaker than that of HIF-1α/2α. To further clarify the mechanism of cell death, we intervened with the ferroptosis inhibitor (Fer-1). The results demonstrated that Fer-1 treatment significantly reversed cell death induced by the overexpression of these proteins (Fig 1), suggesting that ferroptosis was the predominant mode of cell death under hypoxia, and that HIF-1α, HIF-2α, and HILPDA exacerbated this process by promoting ferroptosis.

### Inhibition of HILPDA alleviated hypoxia-induced ferroptosis of NGEC and HIEC

**Fig 2:**
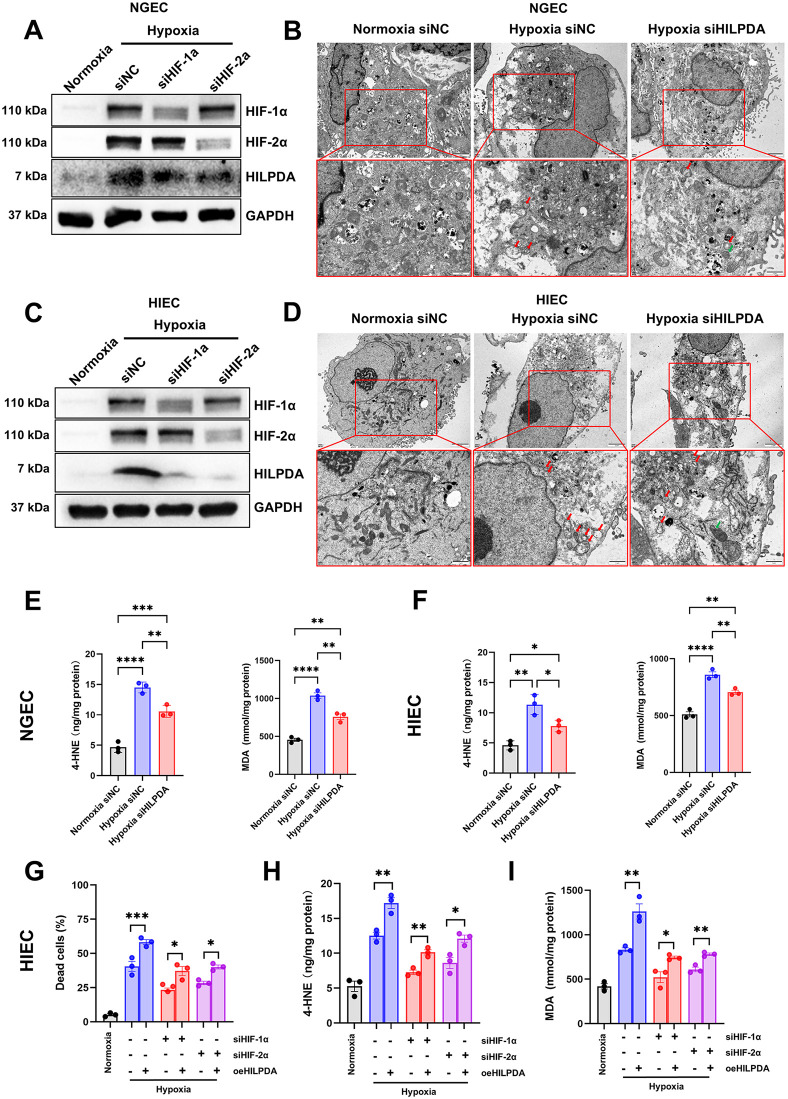
Inhibition of HILPDA alleviated cellular ferroptosis caused by hypoxia. For knockdown experiments, siRNAs for negative control (NC), HIF-1α, HIF-2α, or HILPDA were transfected into NGEC and HIEC under normoxic conditions for 24 h, after which the cells were exposed to hypoxia (1% O_2_) for 48 h. (A, C) The levels of HIF-1α, HIF-2α, and HILPDA assessed by western blot. GAPDH as an internal control. (B, D) Representative images of transmission electron microscopy (scale bar, overview 2 μm, inset 1 μm). Normal mitochondria of NGEC and HIEC are marked with green arrows and mitochondria with the characteristics of ferroptosis are marked with red arrows. A representative of at least three independent experiments is shown. (E, F) 4-hydroxynonenal (4-HNE) and malondialdehyde (MDA) detection. HIEC was co-transfected with siHIF-1α or siHIF-2α (or a non-targeting control, siNC) together with either a HILPDA overexpression plasmid or an empty vector. After 24 h under normoxia, cells were exposed to 48 h of hypoxia. (G) Cell death rate by trypan blue staining. The levels of 4-HNE (H) and MDA (I). The data are presented as the mean ± standard errors of the mean. *p<0.05, **p<0.01, and ***p<0.001.

To validate the morphological features of ferroptosis, we observed mitochondrial structures using TEM. Under hypoxic conditions, NGEC and HIEC cells exhibited mitochondria with increased membrane density and reduced or diminished cristae (Fig 2B, 2D), which were typical characteristics of ferroptosis. However, HILPDA inhibition significantly mitigated these morphological alterations (Fig 2B, 2D and Supplementary S1 Fig). Additionally, by detecting the lipid peroxidation marker 4-HNE and MDA, we found that its levels were significantly elevated in the hypoxia group, while inhibition of HILPDA effectively reduced 4-HNE and MDA levels (Fig 2E, 2F). These results further confirmed that inhibition of HILPDA alleviated hypoxia-induced ferroptosis by mitigating lipid peroxidation.

Subsequently, we examined HIF-1α/2α regulation of HILPDA. The results showed that the expression of HIF-1α, HIF-2α, and HILPDA was significantly upregulated in the hypoxia siNC group (Fig 2A). Notably, knockdown of HIF-1α and HIF-2α using siRNA also led to a reduction in HILPDA expression (Fig 2A), which confirmed that HILPDA was a downstream protein regulated by HIF-1α/2α. Finally, we knocked down HIF-1α/2α while overexpressing HILPDA in NGEC to evaluate the role of HILPDA in HIF-1α/2α-mediated ferroptosis under hypoxia. HILPDA overexpression effectively reversed the attenuation of hypoxia-induced cell death and lipid peroxidation resulting from HIF-1α/2α knockdown (Fig 2G–2I), confirming HILPDA as a key downstream effector of HIF-1α/2α in this pathway.

### Inhibition of HILPDA significantly decreased the abundance of PUFA-PEs and PUFA-PCs

**Fig 3:**
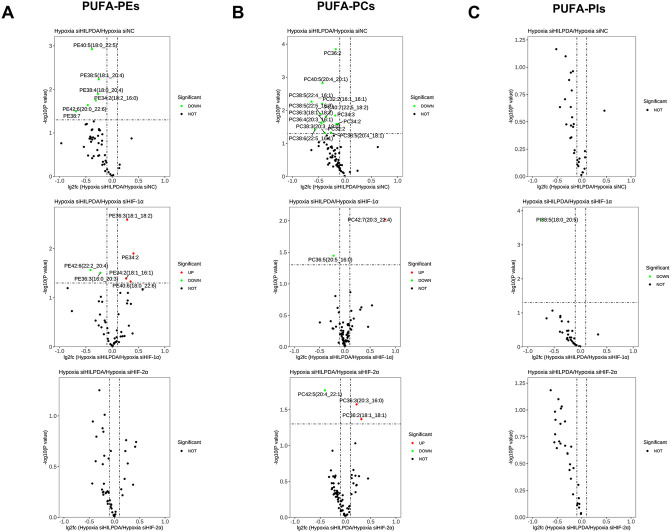
Except for the obvious difference between HIF-1α and HILPDA in regulating PUFA-PEs, there was no significant difference between HIF-1α/2α and HILPDA in the regulation of PUFA-PLs. SiRNAs for negative control (NC), HIF-1α, HIF-2α, or HILPDA were transfected into HIEC under normoxic conditions for 24 h, after which the cells were exposed to hypoxia (1% O_2_) for 48 h. Volcano plots showing the changes of PEs (A), PCs (B), and PIs (C) containing PUFA between the indicated groups (n = 3). PUFA: polyunsaturated fatty acids, PE: phosphatidylethanolamine, PC: phosphatidylcholine, PI: phosphatidylinositol, PLs: phospholipids.

**Fig 4:**
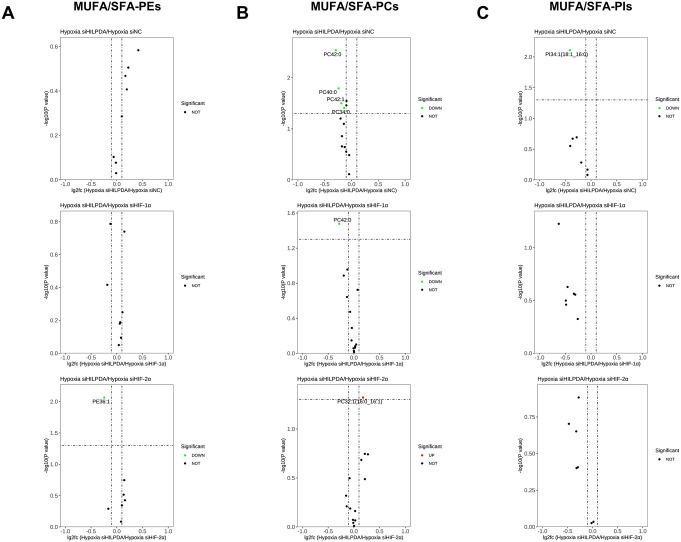
Inhibition of HILPDA exhibited no significant change in the abundance of MUFA/SFA-PEs and PIs. Volcano plots showing the changes of PEs (A), PCs (B), and PIs (C) containing MUFA/SFA between the indicated groups (n = 3). SFA: saturated fatty acids, MUFA: monounsaturated fatty acids. PE: phosphatidylethanolamine, PC: phosphatidylcholine, PI: phosphatidylinositol.

Given that ferroptosis is fundamentally driven by peroxidation of membrane PLs, especially PUFAs containing PEs, PCs, and PIs, we conducted a lipidomic analysis. This analysis aimed to evaluate the impact of HILPDA knockdown on the abundance of PUFA-PEs, PUFA-PCs, and PUFA-PIs, alongside their monounsaturated and saturated counterparts. The results demonstrated that suppressing HILPDA expression led to a significant decrease in the abundance of PUFA-PCs, with a maximum reduction of 13 species observed, which was followed by a decline of 6 species of PUFA-PEs. While PUFA-PIs exhibited no significant decline. Among monounsaturated and saturated fatty acids (MUFA/SFAs), 4 MUFA/SFA-PCs (PC42: 0, PC40: 0, PC42: 1, and PC34: 0) were decreased and PI34: 1 (18: 1_16: 0) was decreased, whereas no significant change was observed in MUFA/SFA-PEs (Figs 3 and 4). From the above results, it was obvious that knocking down HILPDA reduced the abundance of PUFA-PEs and PUFA-PCs.

In our previous study, knockdown of HIF-1α and HIF-2α has been shown to downregulate PUFAs and increase MUFA/SFAs to attenuate hypoxia-induced ferroptosis of cells [10]. This prompted us to investigate whether the observed regulation of lipid metabolism by HIF-1α and HIF-2α was achieved through HILPDA. Consequently, we compared the metabolic effects of HILPDA knockdown with those of HIF-1α/2α knockdown. Compared to knockdown of HIF-1α, knockdown of HILPDA reduced PE42: 6 (22: 2_20: 4), PE36: 3 (16: 0_20: 3), PC36: 5 (20: 5_16: 0), and PI38: 5 (18: 0_20: 5), but elevated PE36: 3 (18: 1_18: 2), PE34: 2, PE34: 2 (18: 1_16: 1), PE40: 6 (18: 0_22: 6), and PC42: 7 (20: 3_22: 4). In addition, there was no significant change in MUFA/SFA-PEs and MUFA/SFA-PIs, and only PC42: 0 was reduced. Compared to knockdown of HIF-2α, knockdown of HILPDA reduced PC42: 5 (20: 4_22: 1) and elevated PC36: 3 (20: 3_16: 0) and PC36: 2 (18: 1_18: 1), with no significant change in PUFA-PEs and PUFA-PIs. In MUFA/SFA, knockdown of HILPDA reduced PE36:1 and elevated PC32: 1 (16: 0_16: 1) compared to knockdown of HIF-2α with no significant difference in MUFA/SFA-PIs (Figs 3 and 4). In summary, the regulatory effect of HILPDA knockdown on lipid metabolism was lesser pronounced than that of HIF-1α/2α knockdown, suggesting that HIF-1α/2α may only partially rely on HILPDA to regulate lipid metabolism pathways.

### HILPDA promoted hypoxia-induced ferroptosis through LPCAT3

**Fig 5:**
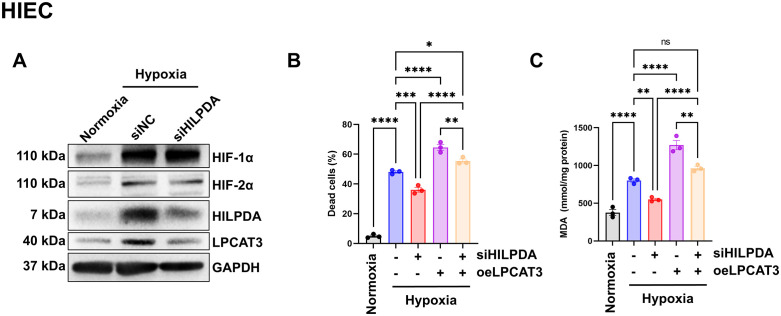
HILPDA promoted hypoxia-induced ferroptosis through LPCAT3. SiRNAs for negative control (NC) or HILPDA was transfected into HIEC under normoxic conditions for 24 h, after which the cells were exposed to hypoxia (1% O_2_) for 48 h. (A) The levels of HIF-1α, HIF-2α, HILPDA, and LPCAT3 assessed by western blot. GAPDH as an internal control. HIEC was co-transfected with HILPDA-targeting siRNAs or siNC together with either LPCAT3 overexpression plasmids or corresponding empty vectors for 24 h under normoxia, after which the cells were exposed to hypoxia (1% O_2_) for 48 h. (B) Cell death rate by trypan blue staining. (C) Malondialdehyde (MDA) detection. The data are presented as the mean ± standard errors of the mean. A representative of at least three independent experiments is shown. *p<0.05, **p<0.01, ***p<0.001, and ****p<0.0001. ns: not significant.

LPCAT3 is a key enzyme that preferentially incorporates PUFAs into membrane phospholipids, particularly at the sn-2 position of PCs and PEs. Given this function, our observation in Fig 3 that HILPDA knockdown under hypoxic conditions primarily reduced PUFA-PE and PUFA-PC levels—without affecting PUFA-PI levels—strongly suggested the potential involvement of LPCAT3, rather than ACSL4, as ACSL4 functions upstream by activating PUFAs for incorporation into all phospholipid classes, including PI. Therefore, in Fig 5, we specifically investigated whether HILPDA mediated ferroptosis via regulation of LPCAT3. The results showed that HILPDA knockdown under hypoxic conditions markedly reduced LPCAT3 protein expression, without affecting HIF-1α and HIF-2α levels (Fig 5A). Furthermore, LPCAT3 overexpression significantly attenuated the inhibitory effect of HILPDA knockdown on hypoxia-induced ferroptosis (Fig 5B, 5C).

## Discussion

This study elucidated the critical role of the HIF-1α/2α-HILPDA-LPCAT3 axis in driving hypoxia-induced ferroptosis through the modulation of lipid metabolism, particularly PUFA containing PLs, in NGEC and HIEC. HIF-1α/2α are known to activate pathways that enhance cellular survival under hypoxia [5]. However, under prolonged or severe hypoxia, regulated cell death pathways may prevail [6].

We found that overexpression of HIF-1α/2α and HILPDA significantly exacerbated hypoxia-induced cell death, which could be alleviated by Fer-1. Furthermore, knockdown of HILPDA significantly reduced hypoxia-induced 4-HNE and MDA production and alleviated hypoxia-induced mitochondrial damage with characteristics of ferroptosis. The above results demonstrated that hypoxia-induced cell death in NGEC and HIEC was mainly ferroptosis, which could be alleviated by HILPDA inhibition.

HILPDA has been implicated in lipid droplet formation and fatty acid storage and emerged as a downstream effector of HIF-1α/2α [23], as evidenced by reduced HILPDA expression following HIF-1α/2α knockdown. However, its role in ferroptosis remains unclear. The regulation of ferroptosis by HILPDA appears to vary depending on the specific tissue type, resulting in variable outcomes across different tissues. In hepatocellular carcinoma, hypoxia-induced HILPDA has been shown to protect cells from oxidative damage by promoting the transfer of PUFAs from membrane phospholipids to lipid droplets, thereby sequestering these oxidizable substrates away from cellular membranes and reducing their susceptibility to ROS-induced peroxidation [29]. Similarly, in nasopharyngeal carcinoma, HIF-1α suppresses ferroptosis by upregulating HILPDA and enhancing lipid droplet accumulation [31]. While others proposed that HILPDA promotes ferroptosis by promoting the abundance of PUFA-PLs in clear-cell carcinomas [25]. In this study, our results demonstrated that HIF-1α/2α mediated hypoxia-induced ferroptosis in a HILPDA-dependent manner. Functionally, the observed reduction in PUFA-PEs and PUFA-PCs following HILPDA knockdown underscored its function in sustaining PUFA-PLs pools, which were essential substrates for lipid peroxidation. Moreover, our findings demonstrated that HILPDA knockdown resulted in a little variation in PUFA-PEs without significant difference in PCs and PIs compared to HIF-1α knockdown. In comparison to the knockdown of HIF-2α, HILPDA knockdown resulted in negligible differences in PEs, PCs, and PIs. The incorporation of PUFAs into membrane PLs, a critical determinant of ferroptosis susceptibility, is largely governed by ACSL4 and LPCAT3 [32]. ACSL4 primarily catalyzes the conversion of PUFAs into PUFA-CoAs. These activated PUFA-CoAs serve as common substrates for the subsequent biosynthesis of various PLs, including PEs, PCs, and PIs [32,33]. LPCAT3 specifically catalyzes the esterification of PUFAs into the sn-2 position of lysophospholipids, thereby enriching cellular membranes with peroxidation-prone PEs and PCs [15]. Our observation that HILPDA knockdown under hypoxic conditions selectively reduced PC and PE levels—without affecting PUFA-PI levels—strongly suggested the specific involvement of LPCAT3, rather than ACSL4. Thereby, we further investigated whether HILPDA regulated ferroptosis through LPCAT3. The results indicated that HILPDA knockdown under hypoxic conditions significantly downregulated LPCAT3 protein expression, and overexpression of LPCAT3 significantly attenuated the inhibitory effect of HILPDA knockdown on hypoxia-induced ferroptosis. These findings demonstrated that HILPDA promoted hypoxia-induced ferroptosis through LPCAT3, which enriched PUFA-PLs and created substrates for lipid peroxidation. In addition to HILPDA, HIF-1α/2α also regulates PUFA through perilipin 2 and G0/G1 switch 2 [25]. Therefore, there may be other pathways by which HIF-1α/2α was involved in the regulation of lipid metabolism to cause the difference between HIF-1α and HILPDA in regulating PUFA-PEs. Overall, HIF-1α/2α relied mainly on HILPDA to regulate PUFA-PLs.

### Conclusions

This study demonstrated that HIF-1α/2α could contribute to lipid peroxidation via HILPDA/LPCAT3 enriched PUFA-PLs, thereby exacerbating hypoxia-induced ferroptosis. These findings advanced our understanding of hypoxia-induced cell death and provided a foundation for targeting lipid metabolism in diseases where ferroptosis played a pivotal role. Targeting the HIF-1α/2α-HILPDA-LPCAT3 axis holds promise for diseases characterized by hypoxia and ferroptosis.

## Supporting information

S1 Fig Quantitative analysis of mitochondria with ferroptotic morphology under transmission electron microscopy (TEM). SiRNAs for negative control or HILPDA were transfected into NGEC and HIEC under normoxic conditions for 24 h, after which the cells were exposed to hypoxia (1% O_2_) for 48 h. For each experimental group, three randomly selected TEM images were analyzed. In each image, the total number of mitochondria was counted, and mitochondria displaying characteristic ferroptotic features were identified. The percentage of mitochondria exhibiting ferroptosis characteristics was calculated per image. The data are presented as the mean ± standard errors of the mean. **p < 0.01, ***p < 0.001, and ****p < 0.0001.
(PPTX)

S2 File Raw images: Original images for western blot. (PDF)

S1 Table Raw data for the bar graphs of MDA, CCK-8, 4-HNE, and trypan blue assays. (XLSX)

## Abbreviations

ACSL4: Acyl-CoA synthetase long-chain family member 4
ALOX5: Arachidonate 5-lipoxygenase
CCK-8: Cell counting kit-8
Fer-1: Ferrostatin-1
HIEC: Normal human small intestinal epithelial cells
HIF-1α: Hypoxia inducible factor-1α
HIF-2α: Hypoxia inducible factor-2α
HILPDA: Hypoxia-inducible lipid droplet-associated protein
HRP: Horseradish peroxidase
LPCAT3: Lysophosphatidylcholine Acyltransferase 3
MDA: Malondialdehyde
MUFA/SFA: Monounsaturated and saturated fatty acid
NC: Negative control
NGEC: Normal human gastric epithelial cells
NOX4: NADPH Oxidase 4
PBS: Phosphate-buffered saline
PCs: Phosphatidylcholines
PEs: Phosphatidylethanolamines
PIs: Phosphatidylinositols
PUFA-PLs: Polyunsaturated fatty acid-phospholipids
TEM: Transmission electron microscopy
4-HNE: 4-Hydroxynonenal
