# Supplementary material for: Lipid droplet associated protein HILPDA promotes hypoxia-induced ferroptosis by driving LPCAT3-mediated polyunsaturated phospholipids enrichment
Source: PLoS One. 2026 Jun 8;21(6):e0350129. doi: 10.1371/journal.pone.0350129 (PMC13245787; doi:10.1371/journal.pone.0350129)
Supplement: S1 Fig — SiRNAs for negative control or HILPDA were transfected into NGEC and HIEC under normoxic conditions for 24 h, after which the cells were exposed to hypoxia (1% O2) for 48 h. For each experimental group, three randomly selected TEM images were analyzed. In each image, the total number of mitochondria was counted, and mitochondria displaying characteristic ferroptotic features were identified. The percentage of mitochondria exhibiting ferroptosis characteristics was calculated per image. The data are presented as the mean ± standard errors of the mean. **p < 0.01, ***p < 0.001, and ****p < 0.0001. (PPTX) [file pone.0350129.s001.pptx]

## Slide 1
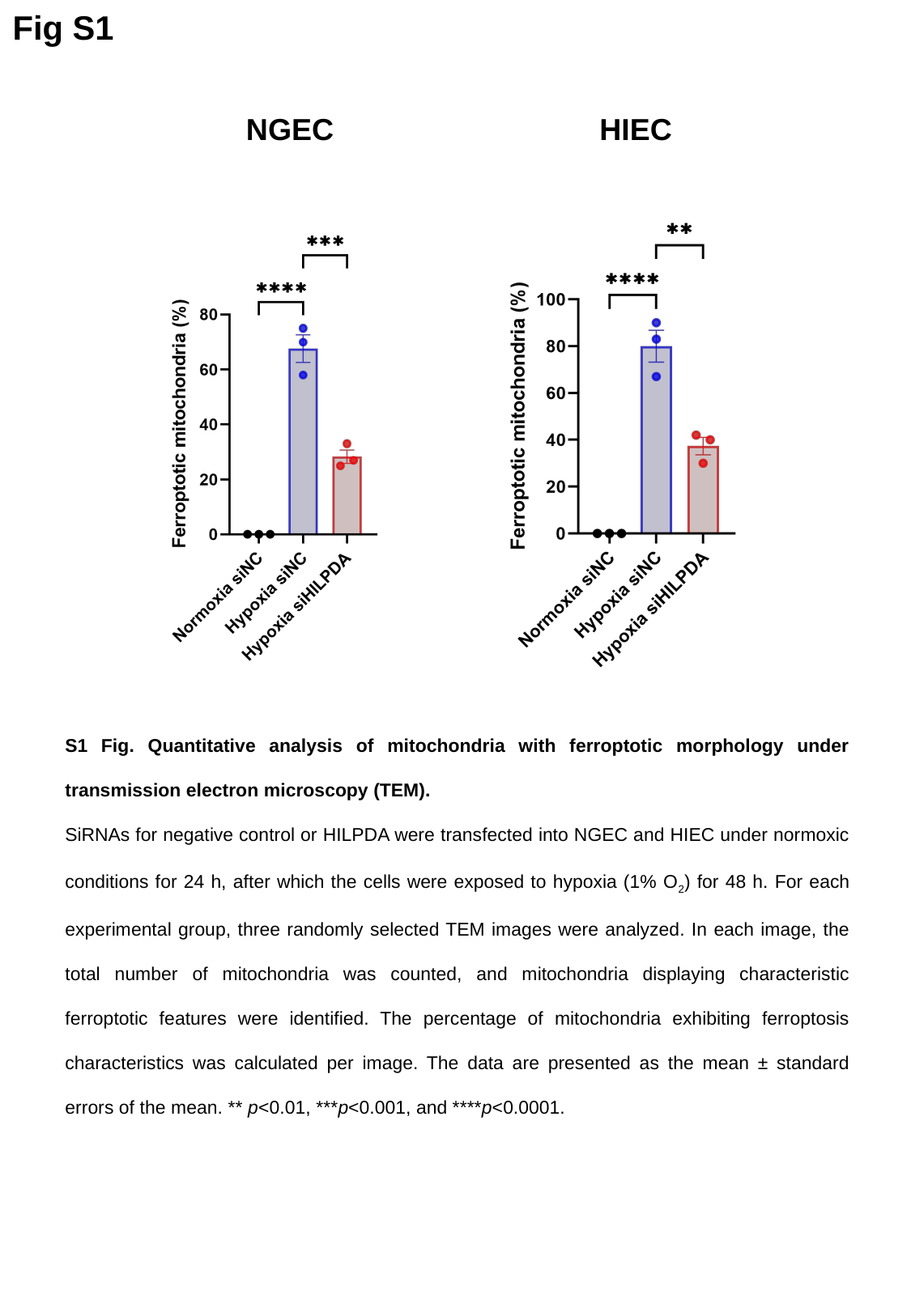

Fig S1
NGEC
HIEC
S1 Fig. Quantitative analysis of mitochondria with ferroptotic morphology under transmission electron microscopy (TEM).
SiRNAs for negative control or HILPDA were transfected into NGEC and HIEC under normoxic conditions for 24 h, after which the cells were exposed to hypoxia (1% O2) for 48 h. For each experimental group, three randomly selected TEM images were analyzed. In each image, the total number of mitochondria was counted, and mitochondria displaying characteristic ferroptotic features were identified. The percentage of mitochondria exhibiting ferroptosis characteristics was calculated per image. The data are presented as the mean ± standard errors of the mean. ** p<0.01, ***p<0.001, and ****p<0.0001.
