# Supplementary material for: Lipid droplet associated protein HILPDA promotes hypoxia-induced ferroptosis by driving LPCAT3-mediated polyunsaturated phospholipids enrichment
Source: PLoS One. 2026 Jun 8;21(6):e0350129. doi: 10.1371/journal.pone.0350129 (PMC13245787; doi:10.1371/journal.pone.0350129)

Orignial Images for Western blot

Protein bands were visualized using an gel imaging system (SinSage Technology Co., Ltd., Beijing, China). To enhance band contrast, the membrane was imaged using the system's 'Low Marker Intensity' mode. If required, the visibility of the marker lanes can be further improved by digitally adjusting the exposure level with Adobe Photoshop.

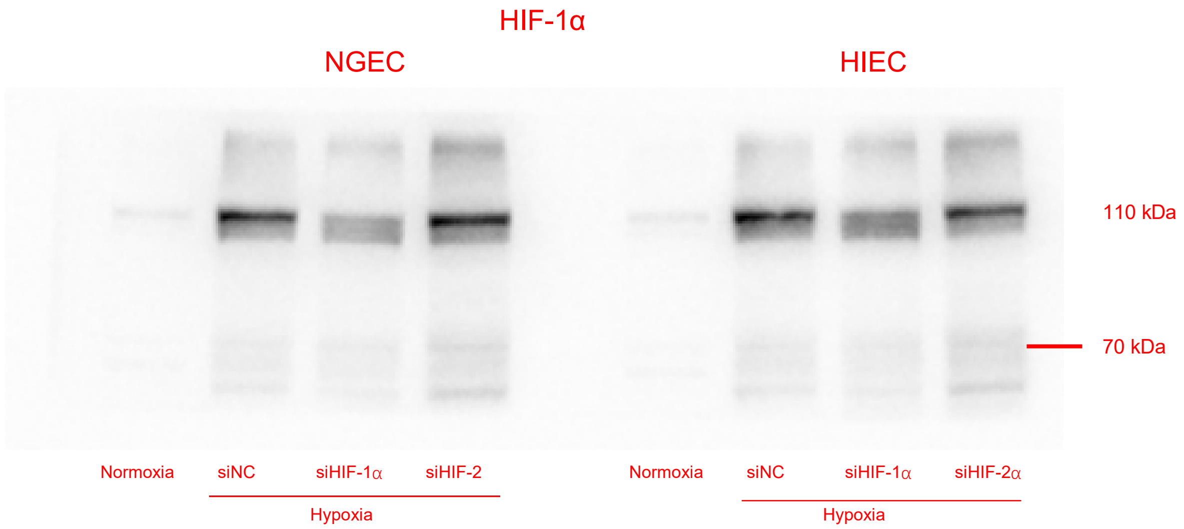

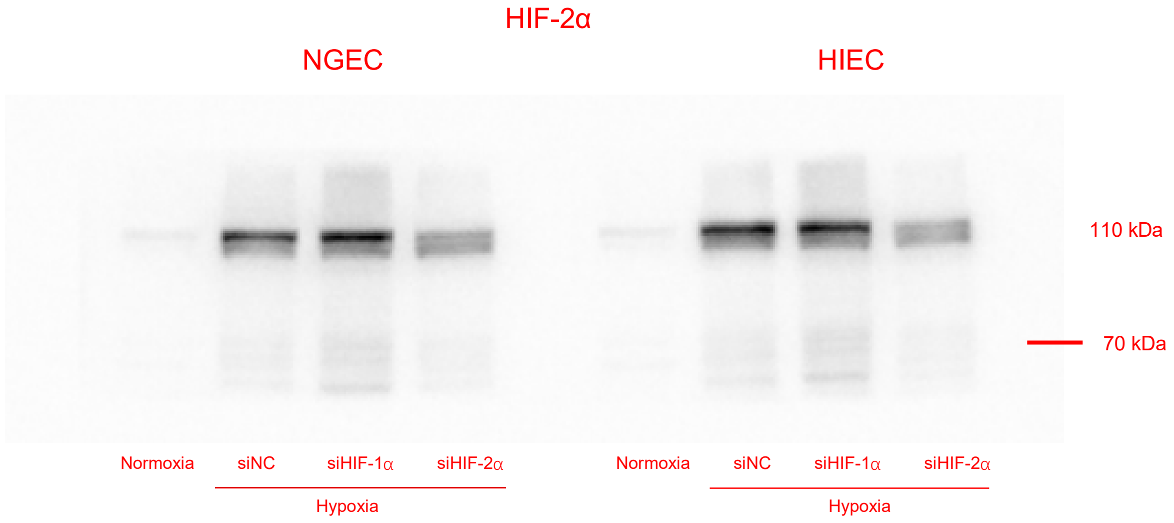

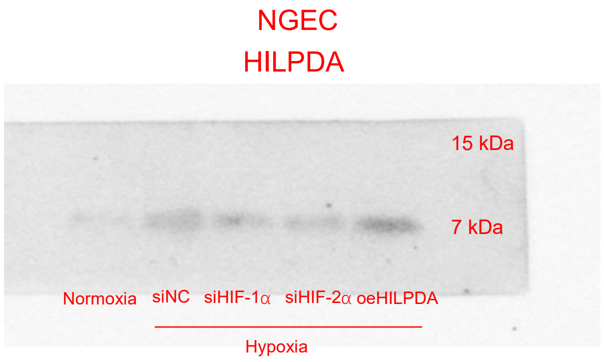

HIEC
HILPDA

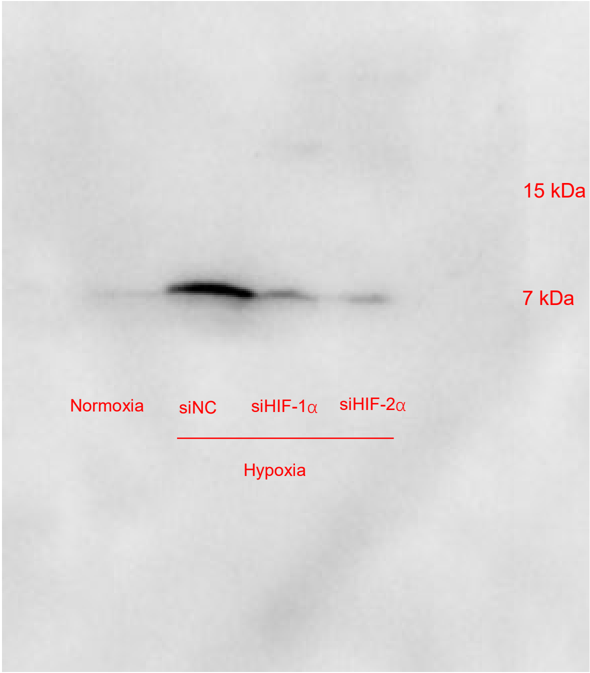

NGEC
GAPDH

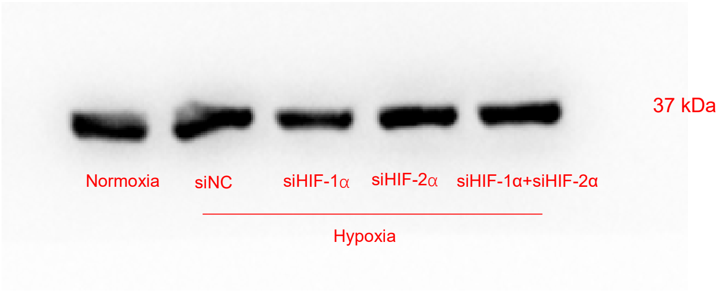

HIEC
GAPDH

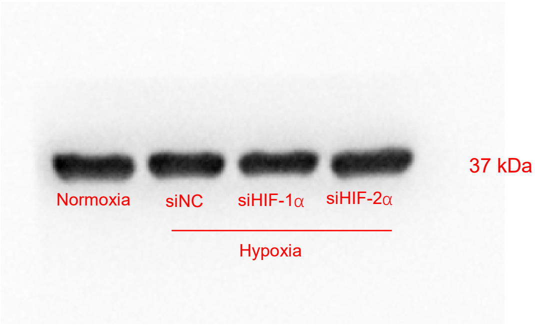

HIEC

HIF-1α

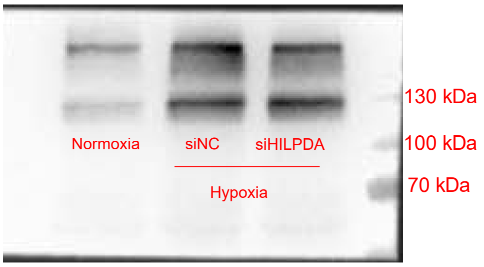

HIF-2α

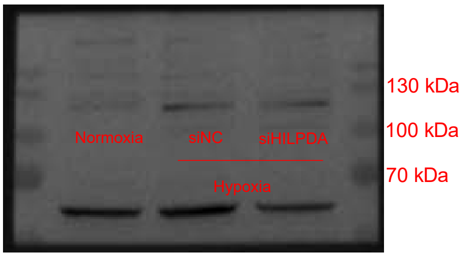

HILPDA

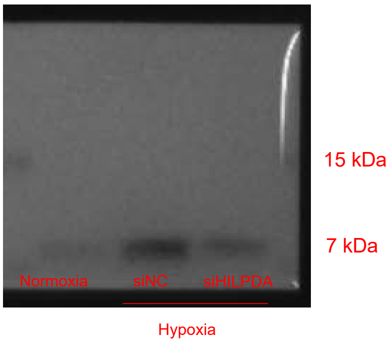

LPCAT3

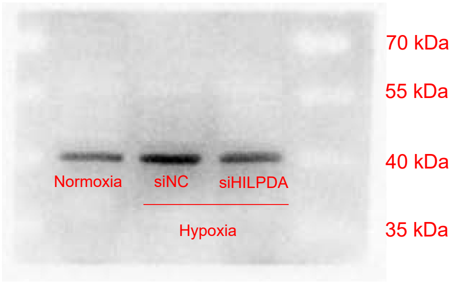

GAPDH

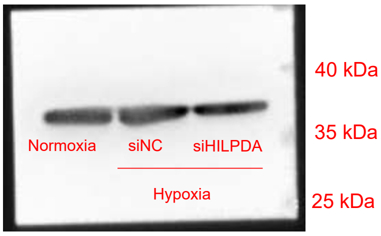

Supplement: S2 File — (PDF) [file pone.0350129.s002.pdf]
